# Doings of Parisian Societies—Gathered from the French Journals by One of Our Correspondents

**Published:** 1861-01

**Authors:** 


ATLANTA

Medical and Surgical Journal.

Vol. VI.] JANUARY, 1861. [No. 5.

### ORIGINAL COMMUNICATIONS.

### ARTICLE I.

*Doings of Parisian Societies—gathered from the French Journals by one of our correspondents.*

ACADEMY OF SCIENCES.

Upon the 10th September, M. Lallemand, in behalf of M. M. Perrin, Duroy and himself, read a memoir on the comparative action of alcohol, the anæsthetic and the carbonated gases upon the cerebrospinal nervous system. After detailing sundry experiments upon dogs he sums up the results as follows:

"The foregoing facts establish a well marked line of demarcation between alcohol and the anæsthetics, chloroform, ether, and amylene on one hand, and the carbonated gases, carbonic acid and carbonic oxide on the other, as regards their physiological action.

"We believe, also, that it is possible to appreciate the nature of the influence produced by these two classes of agents; 1st, alcohol, chloroform, ether and amylene act primitively and directly upon the nervous centres in the substance of which they accumulate. 2d. The carbonated gases exercise primitively a special influence upon the blood; carbonic acid giving to the arterial the color of venous blood, while carbonic oxide alters the state and the physiological properties of the blood globules. It seems to us, then, difficult not to admit that the phenomena of insensibility developed by the inhalation of these gases is but the consecutive and secondary effect of the alteration in the blood. We know that innervation is only brought about upon the condition of the physiological excitation of the nervous system by the blood; we know also that when the blood is not revivified by the contact of oxygen, as in the case of asphyxia from mechanical obstruction to the respiration, or in croup, then occurs a state of anæsthesia which announces imminent danger of the approaching death.

"So the anæsthetics depress and extinguish the functions of the nervous system; their progressive action suspends then the respiration, which is under the influence of the medulla oblongata. They determine then an anæsthesia primitively and an asphyxia consecutively or indirectly.

"Carbonic acid, and carbonic oxide modify the properties of the blood, and impede the keeping up of the innervation. They produce primitively asphyxia or the arrest of hœmatosis and determine a consecutive or indirect anæsthetic.

"*Conclusions*—1st, acohol, chloroform, ether and amylene, act directly and primitively upon the nervous system. 2d, carbonic acid and carbonic oxide, act directly and primitively upon the blood, which they modify; and it is by means of this modification of the blood that they determine secondarily the phenomena of insensibility. These gases then are only *pseudo-anæsthetics*."

*M. Marchal* presented a paper upon a very common, but heretofore underscribed, affection of the gums, which occasions the loss of the teeth, and which he proposes to call

"Expulsive ulitis." This is not a dangerous malady; but in consequence of the constant suffering to which it gives rise, especially at the daily meals, of the bad odor of the breath, which it nearly always causes, of the loss of the teeth, which most frequently are not themselves diseased, of the difficulty of mastication and the pain which accompanies it, this infirmity is a great source of annoyance to those who are affected with it.

"I propose to give it the name of *expulsive ulitis*, recalling by this epithet one of its effects, which is to loosen and then push out the teeth from the alveoli. It presents itself in a variety of forms, being generally suppurating, frequently ulcerating, sometimes vegetating, at others simple in its character.

"There is also a variety in its seat, being first general, or for a time partial, most frequently confined to the tongue-like processes between the teeth; sometimes it is purely inter-alveolar; then the tooth is loosen and nothing more is to be seen upon the surface. There is a difference also in the manner of invasions; sometimes it is by a little phlegmon which maturates, opens itself and leaves the tooth bare; at other times the invasion is by simple inflammation without phlegmon or abscess.

"The causes of expulsive ulitis are, first and foremost hereditary taint; then cold, but especially damp cold, atmosphere; the presence of tartar upon the teeth and gums; pregnancy and nursing; bad state of the stomach, and I would say the hyperœmic gastric irritation, which results from habitual excess in eating.

I believe I have found a remedy, in a manner specific, for this annoying affection in the topical employment of iodine. I do not employ the tincture, unless in exceptional cases, for the alcohol condensing the tissues seems to offer an obstacle to the modifying action or, so to speak, to the penetration of the iodine. I generally use the aqueous solution of varied strength, commencing with the solution of Lugol,* as used for the iodine baths; then gradually increasing the strength of the solution until it becomes quite concentrated.

*M. Baillarger* called the attention of the academy to an interesting point in the history of the general paralysis of the insane; the hypochondriacal delirium considered as one of the symptoms and precursory signs of this terrible malady. At the succeeding session, (Sept. 24th), M. Brierre de Boismont read a paper on "The perversion of the moral faculties and affections in the prodromic period of the general paralysis of the insane, in a medico legal point of view."

"We have seen, with M. Baillarger," says M. Brochin, "that the hypochondriacal delirium was not only a symptom which in most cases serves to diagnosticate the malady at its commencement, but under certain circumstances is a precursory sign which enables us to announce several months, or even years in advance, the probable invasion of the general paralysis, according to the observations of M. Brierre de Boismont, which, as he remarks in his paper, already date back many years, the prodromic period of the malady is still more protracted, and it will be possible from certain signs of perversion of the faculties of the mind, to see still further off its imminence—these perversions sometimes manifesting themselves six, seven or more years before the apparent explosion of the mania. These prodromic phenomena, less distinctly characterized, by reason of their greater distance from the in-

* ℞. Iodine, gr. xij,

Pot. Iodidi. gr. xxiv,

Aquœ fzxss..

Misce.

vasion of the malady than the two special forms of delirium which more nearly precede it and constitute the initial period properly speaking, present the remarkable feature that, in the midst of their apparent diversity, they are able already by certain predominant tendencies to foreshadow to a certain degree the symptoms of the two forms of delirium which should precede and accompany the paralysis."

IMPERIAL ACADEMY OF MEDICINE.

M. Bonisson entertained the academy upon the 2d October, with the report of a highly interesting case of a most unusual character. The subject, a lunatic, blind from cataract, after having been submitted to operation for the removal of the cataract, recovered at once both his sight and his reason. The skill of the surgeon has not only been repaid in this case by the result, always marvelous, and which no one can witness without emotion—the restoration of the sight; but surgery has become in his hands, the instrument of a transformation no less marvelous in the most beautiful and most lofty of the faculties of man—intelligence; and there, where, perhaps, a superficial observer has perceived nothing but a happy coincidence, the learned professor of Montpellier has seized upon and rendered evident to all, a relation which gives to this case the character of one of the most beautiful psychological experiments. In summing up the report, he says:

"In view of these daily changes in the mental state of the patient it is not, we believe, overleaping the bounds of legitimate induction to attribute them to the restoration of the sense of sight. The *post hoc ergo propter hoc*, appears to us here demonstrated by the natural relations of the cause and the effect. Sensation and thought are the two extremes of a psychological affiliation of which our lunatic has furnished an example. The sensation has stimulated the thought, as electricity stimulates nervous action, and we may add, the patient found himself in the condition most favorable for this result. The insanity was not of an inveterate character, and the worn-out sensitive organ was made to produce the most active impressions.

"Is not the sight the sense which multiplies impressions to the highest degree, and which establishes the largest communications with the exterior world? The sense of sight, says Maine de Biran, predominates in the human organism; very closely situated to the cerebral centre, it possesses the distinctive character of communicating to the latter its proper vibrations, and of impressing so directly upon the mental reproductiveness, the images of which it furnishes the ground-work and the first materials. This remark seems to have been made expressly for the case which has furnished the subject of these reflections: it is, however, but the embodiment of an opinion accepted by all philosophers upon the power which the sense of sight exercises over the formation of ideas. Descartes and Locke, notwithstanding their doctrinal differences, are agreed upon this point. Locke compares the human intelligence to a darkened chamber, pierced with windows by which light enters, and that which admits most is the vismal window. Our patient has received through this opening the impressions which have brought him in communication with the exterior world. The impressions are so varied and multiplied, that there is no room for astonishment at the effect they have produced. It is doubtful whether the restoration of any other sense than the sight could so promptly have produced the same result."

SOCIETY OF SURGERY.

Upon the 19th September, M. Vernenil, remarked upon a paper of Mr. Bitot of "L'ecole secondaire de Bordeaux," entitled "*Fibroplastic tumor, Middle tarsal disarticulation with conservation of the scaphoid; simultaneous section of the tendo-achillis; superiority of this operation over that of Chopart.*" The case was that of a boy, of 13 years, affected with a fibrous tumor of the left foot, occupying the metatarsus, especially the second bone of this region. All the anterior part of the foot having to be sacrificed, M. Bitot decided to resort to the middle tarsal amputation, preserving the scaphoid, which appeared to him to be sound. He made in the first place, the subcutaneous section of the tendo-achillis, with a tenatome introduced by a small cutaneous incission from the inner aspect of the foot, and then proceeded to the operation, substantially that of Chopart. Notwithstanding some local and general disturbance, the cicatrization was complete at the end of 40 days. During the treatment no attention was paid to the divided tendon, but the dressings were applied precisely as though this section had not been made.

Nine months after the operation, the stump presented the following appearances: the line of cicatrix was bent and slightly movable at the centre. In the ordinary position the stump rests upon the sole throughout its plantar surface, measuring 12 centimetres (4¾ inches), in length; but the pressure is principally upon the posterior, three-fourths of this surface. There is not the slightest latteral deviation; the sole of the short shoe, that the patient has worn for more than five months, is still very uniform, no more worn upon one side, than the other. The stump is flexed and extended with the greatest facility; the extension, however, being quicker and of wider range, than the flexion. The patient walks with ease; almost without lameness.

"If the success has been complete," says M. Bitot, "it is because of the conservation of the scaphoid, which plays an important part in standing and walking. This bone removed, the internal antero-posterior arch of the foot dissappears entirely, and the foot is seen to turn outwards. Its conservation leaves intact the action of the tibialis posticus, whose office is to elevate the inner border of the foot. It may, perhaps, be objected that this muscle being also an extensor, in seeking to prevent the outward deviation of the stump, we favor the deviation backwards, which is more to be dreaded than the other; but we have happily, in the section of the tendo-achillis, a means very simple and for the most part efficacious, of obviating this inconvenience.

"No one contests the safety of this subcutaneous operation, but its efficacy is not so readily admitted, for the reason that it has only been practiced consecutively; that is to say, at a period more or less remote from the amputation. *To be really useful, this section ought to be primitive; in other words, made at the same time with the operation, immediately before or immediately after it.*"

*Conclusions*—"1st, That the middle tarsal disarticulation with conservation of the scaphoid is preferable to the amputation of Chopart. 2d, That in the tarsal disarticulation, the *immediate* section of the tendo-achillis is at least always useful, if not indispensable, for complete success; and that the *consecutive* section has not the same value. 3d, That the patient after the operation ought to extend the limb, and rest it upon the calf of the leg."

M. Vernenal observes: "the amputation of Chopart destroys the insertions of all the muscles which move the tibio-tarsal articulation, the gastrocnemius and solens excepted; which remaining without antagonism, are able during the cicatrization, easily to draw up the os-calcis and produce a deviation which becomes permanent, because of the adhesions that the deep tendons form with the grooves of that bone. Having myself, heretofore, observed these adhesions, I am inclined to regard them as the cause of the deviation, whilst they are, perhaps, but the natural effect of the cicatrization in a bad position produced by the muscles of the calf, to which I deny the participation generally admitted.

"I have elsewhere said, that in the old organic affections, the foot was, at the moment of amputation, in a state of forced extension; hence the posterior extremity of the os-calcis is already elevated, and the drawing up of the heel in part accomplished. This mal-position is not corrected during the cicatrization, and whenever this is achieved, the calcaneum is solidly maintained in an oblique position with its anterior extremity depressed. It is well known that the dressings, the cushions, the inclined planes, &c., are unable to counteract the drawing up of the heel; the section of the tendo-achillis itself is fruitless, because the deep tendons, if they have not produced the difficulty, nevertheless maintain and render it permanent.

"Theory promises an entirely different result if the tendon is divided primitively. The first row of the tarsus is only united to the leg by the ligaments of the tibio-tarsal articulation. If this, at the moment of operation, is in a state of forced extension, it is easily restored and flexed after having divided the tendo-achillis. The tendons of the anterior muscles of the ankle, if they have not been divided too high, are able to contract adhesions with the head of the astragalus which comes in contact with them. By the fact itself of this flexion the opposing tendons (the peronii-posterior tibial and flexors of the toes), are retracted in their sheaths and contract adhesions at a point less favorable for the extension of the foot which it is sought to prevent.

"If, as M. Bitot recommends, at the same time, care is taken to rest the leg upon the calf, the two ends of the tendo achillis separate, and after the reunion by an intermediate fibrous callus, the tendon, elongated two or three centimetres, acts less powerfully upon the os-calcis and loses its tendency to elevate the posterior end of that bone. Lastly we may add that the stump of the tarsus being, so to speak, floating in consequence of the destruction of all its tendinous insertions, may be easily maintained in a state of flexion, that is to say, in a sloping position, by the reunion of the anterior and posterior flaps, and by the dressings arranged for this end."

M. Huguier insisted that the section of the tendo-achillis is not always necessary. A patient for whom he had amputated nine years ago, died recently and the society had the opportunity of seeing that the stump presented no deviation backwards.

M. Chassaignac thought that the opinions of M. Bouvier ought to be modified by the discussion. For his own part, he considered the amputation of Chopart as an excellent operation. Cases have been cited of patients who walk well without the section of the tendon; others walked upon the front of the os-calcis. The conclusion of the discussion ought to be, that it is proper to perfect the operation of Chopart by tenotomy, anchylosis, &c., and not to reject it from surgical practice, inasmuch as it is an operation which gives a very good result. M. Bouvier had said, that the hospitals were encumbered with persons amputated by the method of Chopart; this proves that it is often followed by recovery, which cannot be said of many other amputations.

